# Cognitive Abilities of Dogs with Mucopolysaccharidosis I: Learning and Memory

**DOI:** 10.3390/ani10030397

**Published:** 2020-02-28

**Authors:** Lena Provoost, Carlo Siracusa, Darko Stefanovski, Yan Che, Mingyao Li, Margret Casal

**Affiliations:** 1 Small Animal Behavior Service, Department of Clinical Sciences and Advanced Medicine, University of Pennsylvania, 3900 Spruce Street, Philadelphia, PA 19104, USA; 2 Department of Clinical Sciences and Advanced Medicine, University of Pennsylvania, New Bolton Center, Kennett Square, PA 19348, USA; sdarko@vet.upenn.edu; 3 Gene Therapy Program, Department of Medicine, The Perelman School of Medicine, University of Pennsylvania, 3400 Civic Center Blvd., Philadelphia, PA 19104, USA; yanche@pennmedicine.upenn.edu; 4 Department of Biostatistics and Epidemiology, The Perelman School of Medicine, University of Pennsylvania, 3400 Civic Center Blvd., Philadelphia, PA 19104, USA; mingyao@pennmedicine.upenn.edu; 5 Section of Medical Genetics, Department of Clinical Sciences and Advanced Medicine, University of Pennsylvania, 3900 Spruce Street, Philadelphia, PA 19104, USA; casalml@vet.upenn.edu

**Keywords:** MPS I, dog, cognition, memory, learning

## Abstract

**Simple Summary:**

Lysosomes are cell organelles that contain enzymes that break down large molecules to be recycled or discarded. When lysosomal enzymes fail to perform this function, molecules become trapped and cause cellular destruction. Mucopolysaccharidosis I (MPS I) is a rare disease that occurs in dogs and humans due to a deficiency of the lysosomal enzyme, alpha-L-iduronidase. Humans affected with MPS I experience mild to severe clinical signs in facial features, skeletal changes, cognitive decline, and heart, liver, and respiratory disease. Similarly, MPS I in dogs also cause facial changes, musculoskeletal degeneration, spinal cord compression, and heart and liver disease. However, the cognitive ability in dogs affected with MPS I has not been investigated. The purpose of this pilot study was to determine the feasibility of conducting cognitive tests on MPS I affected dogs and their cognitive abilities. Three groups of dogs were tested: MPS I untreated, MPS I treated, and clinically normal. Dogs were successfully trained to perform the cognitive tests. Differences in their ability to reach the criterion was evident in attention oddity and scent discrimination tests. This study found cognition testing of dogs affected with MPS I to be feasible and recommend future studies focus on a single cognitive domain at a time.

**Abstract:**

Mucopolysaccharidosis I (MPS I) results from a deficiency of a lysosomal enzyme, alpha-L-iduronidase (IDUA). IDUA deficiency leads to glycosaminoglycan (GAG) accumulation resulting in cellular degeneration and multi-organ dysfunction. The primary aims of this pilot study were to determine the feasibility of cognitive testing MPS I affected dogs and to determine their non-social cognitive abilities with and without gene therapy. Fourteen dogs were tested: 5 MPS I untreated, 5 MPS I treated, and 4 clinically normal. The treated group received intrathecal gene therapy as neonates to replace the *IDUA* gene. Cognitive tests included delayed non-match to position (DNMP), two-object visual discrimination (VD), reversal learning (RL), attention oddity (AO), and two-scent discrimination (SD). Responses were recorded as correct, incorrect, or no response, and analyzed using mixed effect logistic regression analysis. Significant differences were not observed among the three groups for DNMP, VD, RL, or AO. The MPS I untreated dogs were excluded from AO testing due to failing to pass acquisition of the task, potentially representing a learning or executive function deficit. The MPS I affected group (treated and untreated) was significantly more likely to discriminate between scents than the normal group, which may be due to an age effect. The normal group was comprised of the oldest dogs, and a mixed effect logistic model indicated that older dogs were more likely to respond incorrectly on scent discrimination. Overall, this study found that cognition testing of MPS I affected dogs to be feasible. This work provides a framework to refine future cognition studies of dogs affected with diseases, including MPS I, in order to assess therapies in a more comprehensive manner.

## 1. Introduction

Lysosomal storage diseases affect many mammalian species, including, humans, dogs, cats, cattle, goats, sheep, mice, and monkeys [1,2,3,4,5,6]. These disorders are the result of deficient hydrolytic enzyme activity or associated accessory proteins [1]. (Please see Table S1 in Supplementary Materials for complete list of lysosomal storage diseases that affect both dogs and humans). Mucopolysaccharidosis I (MPS I), a lysosomal disease, occurs in approximately 1 out of 100,000 live human births [7]. Like most storage diseases, MPS I is inherited as an autosomal recessive trait [8,9]. Those affected with MPS I have an alpha-L-iduronidase (IDUA) deficiency resulting in glycosaminoglycan (GAG) accumulation within lysosomes, primarily dermatan and heparin sulfates. This inability to catabolize GAGs leads to primary and tertiary damage, such as cell degradation and multi-organ dysfunction.

In humans, clinical signs associated with MPS I occur on a spectrum and have historically been described as three syndromes: Hurler (severe), Scheie (mild), and Hurler–Scheie (intermediate). However, due to the overlap of these syndromes, the disease is currently classified as either severe or attenuated [9]. Children affected with severe forms succumb to the disease by 10 years of age [9]. These patients experience multisystem dysfunction including progressive cognitive impairment, morphological brain abnormalities, skeletal deterioration, cardiopulmonary disease, hepatosplenomegaly, and sensory deficits in hearing and vision [7,10,11]. Individuals affected with attenuated forms experience similar multi-organ dysfunction; however, clinical signs are often varied and less severe, with most children surviving into adulthood. These patients have been described as having cognitive deficits, sleep problems, and develop behavioral disorders during childhood and adolescence [7,12]. Magnetic resonance imaging (MRI) studies of the central nervous systems (CNS) of MPS I affected children show enlarged perivascular spaces, white matter lesions, hydrocephalus, cerebral cortical atrophy, spinal canal stenosis, and dystosis multiplex [13,14]. Historically, MPS I–VII patient data has been combined resulting in conflicting relationships between MRI findings and cognitive deficits [15,16].

A diagnosis of MPS I in humans is made when low alpha-L-iduronidase activity is found in fibroblasts, leukocytes, serum, or blood [17,18]. Since clinical signs may not appear until 12 months or later, diagnosis is often delayed; though, prenatal screens are available for those with familial risks [9,19]. A lag in the diagnosis of severe forms of MPS I lead to delayed treatment resulting in sub-optimal treatment outcomes [19]. Treatment of MPS I is aimed at alleviating clinical signs and preventing progression through enzyme replacement therapy (ERT), hematopoietic stem cell transportation (HSCT), or both [9,20]. HSCT using bone marrow or umbilical cord blood is reserved for humans affected with the severe forms of MPS I, as there is a risk of death and morbidity from pulmonary and cardiac complications [9,20]. ERT using recombinant human IDUA (rhIDUA), either intrathecally (IT) or intravenously (IV), has been shown to be efficacious in alleviating mild and intermediate clinical signs [9,21]. A major limitation of IV administration is the inability to pass the blood–brain barrier resulting in little to no benefit on brain pathology or cognitive decline [22]. One way to overcome this challenge is through IT administration. In the dog model of MPS I, IT administration has been shown to reduce GAG levels in cerebral spinal fluid (CSF) [23] and reduce brain pathology [24]. IT therapy in humans is still undergoing clinical trials, though safety and efficacy have been reported [10,25]. A case report of an MPS I human patient treated with IT rhIDUA for spinal cord compression resulted in normal CSF GAG levels, improved stability and gait when walking, and improved ventilation [25]. A second case study of an MPS I attenuated patient treated with IT rhIDUA resulted in increased white matter, increased corpus callosum volume, and improved cognition, particularly memory [10]. Such positive benefits associated with the CNS would not be possible with IV therapy alone.

Neurological, cognitive, and behavioral signs associated with MPS I remain untreated if therapies are unable to target the CNS and therefore negatively affect quality of life. Reported cognitive impairments of MPS I human patients include low intelligence quotients (IQ), poor attention scores, and memory deficits when compared to non-affected individuals [10,26]. Cognition tests of human MPS I patients reflect impaired working memory [26] which may be due to smaller hippocampal volumes [27]. Furthermore, MPS I affected mice show deficits in learning and navigation [28,29], impaired long-term memory and reduced rearing behavior [30]. Despite reports of behavioral and cognitive differences in MPS I affected mice [31] and humans [26], very little has been described in dogs. There are anecdotal reports and personal communications [32] suggestive of behavioral differences in MPS I affected dogs [33]. Studies investigating the treatment efficacy of MPS I affected dogs reported that, aside from proprioceptive deficits, affected untreated dogs exhibited normal behavior, chased balls, and responded to their names [24,34]. To date, there are no studies exclusively dedicated to the behavior or cognitive ability of MPS I dogs despite their use as models for the disease and treatment outcome [24,34,35,36,37]. Additional research may assist clinicians to recognize and question owners regarding behavior traits and cognitive characteristics that may be associated with MPS I.

Expert clinicians estimate that the incidence of canine MPS I could be as low as one affected subject in 100,000 to 500,000 dogs [32]. The authors (M.C.) have clinically evaluated 12 privately owned dogs with naturally occurring MPS I that were presented between 2–6 months of age for stunted growth, abnormal gait, and cloudy corneas [32]. Additional physical exam findings include lax joints that may be fluid filled but not overtly painful, umbilical hernias, ataxia, a heart murmur, facial dysmorphia with prognathism inferior, low set ears and coarse features. Diagnostic workup includes screening for urinary GAGs with a toluidine blue spot test. If positive, confirmatory genetic (blood sample in EDTA tube or cheek swab) and enzymatic (fresh sample with control shipped overnight on ice) testing should be submitted to the PennGen Laboratory, which is able to run the assay. Symptomatic treatment with analgesics, anti-inflammatories, and physiotherapy is recommended [38].

Natural occurring models in dogs, cats, and mice have been used to investigate MPS I disease processes and treatments [35]. However, the MPS I dog model is preferred for IT studies due to their comparable brain morphology to humans and ideal size for therapy administration and imaging [24]. Clinical signs of MPS I affected dogs are analogous to the human intermediate form, which, again, include, skeletal deterioration, joint laxity, hepatosplenomegaly, heart disease, impaired vision, and a lifespan of less than 3 years [24,39]. Radiographs of MPS I affected dogs show joint effusion with secondary degenerative joint disease of multiple joints, lytic areas of the spine, and variable degrees of narrow intervertebral disk spaces with osteophyte formation [39]. Brain MRIs of MPS I affected dogs show that they experience cerebral ventricular enlargement, cortical atrophy, meningeal thickening, as well as thinning and volume loss of the corpus callosum by 12–18 months of age when compared to non affected dogs [24,37]. These are similar changes to those observed in MPS I affected children, providing additional support for use of MPS I dogs as a model [13,14].

Several neuropsychological tests have been developed to measure cognitive abilities of humans to help understand disease progression and diagnose medical conditions. These tests have been adapted for many species, including dogs. For instance, recent research has suggested that dogs suffering from idiopathic epilepsy have spatial working memory deficits that may be helpful in identifying adjunctive treatments [40]. Several cognitive domains in dogs have been investigated, such as visuospatial learning, object discrimination, and attention [41,42]. Some of these domains are used to evaluate higher order brain processes, termed executive functions, which include inhibition, working memory, and cognitive flexibility [43,44]. The frontal lobe, subdivided into prefrontal- and primary motor cortices, plays a major role in executive function [44]. A decline or poor executive function ability indicates cognitive deficits in dogs [45] and humans [46]. Therefore, the primary aims of this study were to determine the feasibility of performing cognition tests on MPS I affected dogs and to determine spatial working memory, long term memory, attention, and executive function abilities. As a pilot study, additional goals were to identify relevant information to refine our protocols including apparatus setup, cognitive task types, and trials. Based on the cognitive deficits observed in human MPS I affected individuals, the authors hypothesized that cognitive deficits would also be observed in dogs affected with MPS I, in addition to sensory deficits associated with disease progression, such as vision and olfaction. It was also hypothesized that the number of cognitive tasks could be reduced but suspected that the number of trials would remain constant. Other than aiming to test a viable model for cognition changes observed in humans and dogs with MPS I, this pilot study seeks to provide a set of useful tests to study the effects of other diseases potentially affecting dog cognition, as well as treatment efficacy, in a more comprehensive manner.

## 2. Materials and Methods

All animals were bred under institutionally approved protocols at the University of Pennsylvania (Philadelphia, PA) and cared for in accordance with the principles outlined in the National Institutes of Health Guide for the Care and Use of Laboratory Animals and the International Guiding Principles for Biomedical Research Involving Animals. The Institutional Animal Care and Use Review Committee approved the study. Three groups of dogs were included for the study: clinically normal dogs (heterozygous for *IDUA* gene), MPS I affected untreated (homozygous recessive for *IDUA*), and MPS I affected treated (homozygous recessive for *IDUA* that was administered IT therapy described below). The dogs were housed in a temperature and humidity controlled environment with a 12-h light/dark cycle. Dogs were fed twice daily regardless of testing schedule and had access to fresh water ad libitum. Dogs were fed a growth diet until the age of 1 year, at which time they were switched to an adult maintenance diet. Same sex pair housing was utilized with one pair being separated during feeding time due to food related aggression. All dogs were provided a rotation of enrichment items when kenneled. The dogs were of mixed breed heritage comprised of Beagle and Plott hound. Heterozygous adult females were artificially inseminated with sperm from heterozygous males to produce affected offspring. All whelps remained with their dams until 8 weeks of age. There were no brother–sister, father–daughter, or mother–son matings. Defined endpoints for the study, in which a dog was euthanized, included more than a 20% weight loss of original body weight, anorexia lasting more than four days not responsive to supportive care, severe loss of mobility, pain or discomfort not responsive to analgesics within 24 h, and untreatable paralysis.

Affected animals were identified by genotyping at birth using 0.5 mls of EDTA blood. The assay reagents were obtained from the Custom Taqman SNP Genotyping Assay provided by Applied Biosystems (Life Technologies, Grand Island, NY, USA). The DNA fragment around the point of possible mutation was amplified by real time PCR using a 15 µL reaction mixture created from 7.5 µL of 2× TaqMan^®^ Universal PCR master mix, no AmpErase^®^ UNG (Applied Biosystems, Life Technologies, Grand Island, NY, USA), 0.375 µL of 40× assay mix consisting of unlabeled PCR primers (forward: GCCCCCTCGCTCTGC; reverse: GTCCCAGCTGAGGTCATAGC), and fluorescence labeled TaqMan^®^ MGB probes (CCCCCCTGCCGCAC, VIC^®^ dye-labeled for wild type, and CCCCCCGCCGCAC, FAM™ dye-labeled for mutant), 6.125 µL of diH_2_O and 1 µL of template gDNA. The cycling conditions utilized mirrored the universal thermal cycling parameters: initial heating step at 95 °C for 10 min, 40 cycles of 15 s at 92 °C for denaturing and 60 s at 60 °C for annealing/extending, and 10 min at 72 °C for final extension. Amplification, detection, and data analysis were performed with an Applied Biosystems ABI 7500 instrument (Life Technologies, Grand Island, NY, USA).

Affected dogs do not produce any enzyme, as the disorder is caused by a null mutation in IDUA [47]. Therefore, the treated dogs required tolerization via enzyme replacement prior to administration of gene therapy at 4 weeks of age [48,49]. Five dogs were treated with 0.58 mg/kg laronidase (Aldurazyme^®^, BioMarin Pharmaceutical Inc., San Rafael, CA, USA) IV at 7 and 14 days of life. At 28 days of life, these 5 dogs were induced with propofol IV and intubated. The area over the cerebellomedullary cistern was clipped and aseptically prepared. One ml of the IDUA vector, at a titer of 1 × 10^12^ GC/kg, was slowly infused intrathecally over 2–3 min. As previously described, the IDUA vector consisted of AAV serotype 9 capsid carrying the chicken beta-actin promoter, cytomegalovirus immediate early enhancer, the human IDUA cDNA, rabbit globulin poly-adenylation, which was flanked by AAV2 terminal repeats [49].

Overall 19 dogs were enlisted for preliminary training which consisted of introducing the dogs to cups, the testing apparatus, and to stand or sit on a mat. Dogs were trained to approach and displace cups using food lures and positive reinforcement; initially the cups were placed on a tray and progressed to the testing apparatus. The dogs were also trained to stand or sit on a mat (placed in front of the apparatus) initially by luring them onto the mat with food rewards, and then pointing to the mat and tossing them a treat once on it. Each dog was trained for 15–20 min once weekly [50] on an average of 2.5 months. Once dogs immediately and willingly approached the apparatus, displaced cups, and positioned onto the mat, they began preference evaluation. Dogs were excluded if after 2 months of preliminary training they continued to jump or walk through the shade and could not position onto the mat. Three dogs (2 clinically normal and 1 MPS I affected untreated) did not pass preliminary training and were excluded. Two MPS I affected untreated dogs were euthanized during preliminary training due to disease progression; 1 developed septicemia and 1 acquired a cranial cruciate ligament rupture and was non-ambulatory. Thus, a total of 14 intact dogs were enrolled in the study for which they completed preliminary training and preference testing over weekly sessions for an average of 2.5 months (see Table 1). A single blinded experimenter (to the groups) performed the training and testing of all dogs in the study.

**Figure 1:**
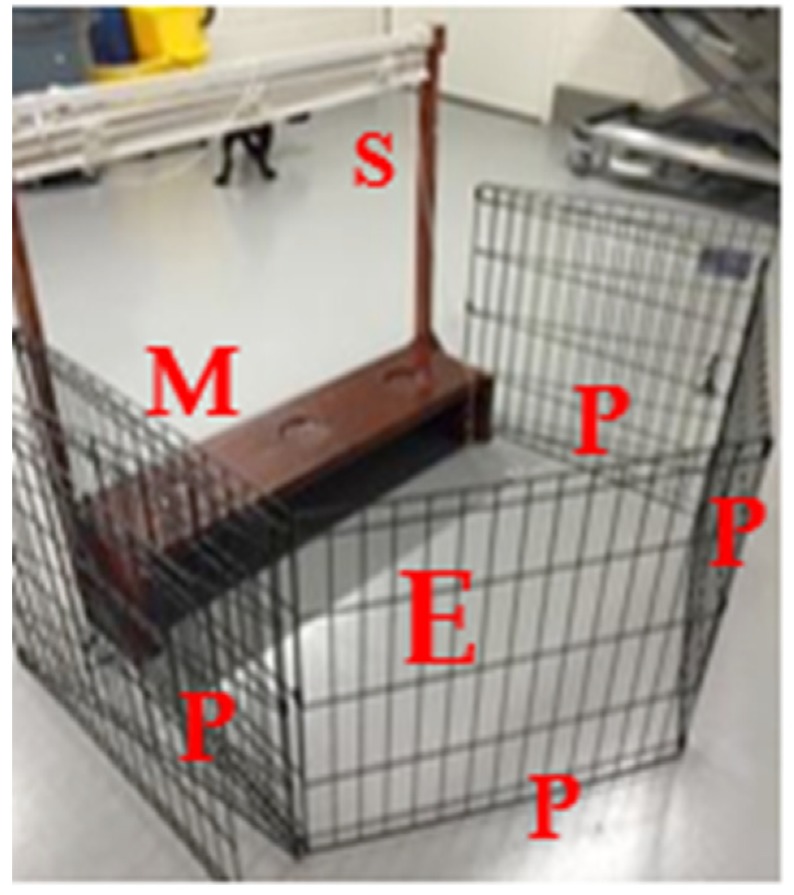
Modified testing apparatus. Dogs were trained to position face forward on the mat (**M**). The experimenter (**E**) remained behind the apparatus within an exercise pen with opaque panels (**P**) (not shown) and a functional shade (**S**). Supplies for each trial were kept underneath the tray of the apparatus.

A modified testing apparatus (Figure 1) was utilized for all tests. Cognitive test procedures were tailored for animals that would eventually experience musculoskeletal disease. Since musculoskeletal disease and gait abnormalities were expected, the testing apparatus needed to accommodate for such physical changes. Therefore, to ensure that procedures could be carried out in a sternal recumbent position (if the dog preferred), the apparatus was 91.4 cm × 95.89 cm × 25.4 cm. It consisted of a drawstring shade to allow the test field to remain out of the subject’s sight and an elevated shelf with three circular wells, 1.27 cm deep and 10.2 cm in diameter, spaced approximately 17.8 cm apart. The dogs were trained to stand or sit face forward on a mat approximately 45.7 cm in front of the apparatus. The experimenter remained separated from the dogs, enclosed behind the apparatus and an opaque fence (an exercise pen with cardboard panels) for the entirety of the session (see Figure 1). During the preparation of each trial, the shade remained closed so that the entire setup of the test field was not visible to the dog. When each trial was then executed, the shade was partially lifted below the level of the experimenter’s shoulder to allow the experimenter to observe the apparatus, while still preventing the dog from making eye contact with the experimenter.

Dogs began preliminary training between two and four months of age to approach a testing apparatus and displace paper cups with their muzzle using positive reinforcement. Aside from their regular diet, Iams^®^ chicken canned food, only single ingredient treats were utilized. Flavor tests of treats were performed with the dogs eating chicken flavor (versus Cheddar cheese, liver, bison, duck, fish) most consistently. Therefore, Purebites^®^ freeze dried chicken and Iams^®^ chicken canned food were used as positive reinforcement. Testing and training sessions were held in the afternoon hours. All testing was performed in a separate room adjacent to the dogs’ kennels.

Cognitive tests began when dogs were between four and seven months of age. Prior to testing, each dog went through a preference evaluation for color, side, and object to utilize features motivating to each dog. A color and side preference (left versus right) evaluation for delayed non-match to position tests using plastic cups (blue versus red) and the apparatus (Table 2). Cups that appeared as dark blue and red to the human eye were used. Dogs have dichromatic vision due to the two types of photo pigments in their cone cells, with absorption maxima at 430 nm and 555 nm [51,52]. In between these two peaks, there is a neutral point, i.e., the wavelength around which dogs are less able to discriminate between two different colors has been determined to be 480 nm for dog vision. Two colors with wavelengths close to the neutral point are difficult to set apart for dogs, especially when they are both on one side of the neutral point. However, when between the wavelengths of the two hues of color there is a large distance in which dogs can differentiate them. Colors perceived as different hues of blue by humans have a wavelength of about 430–486 nm, while colors perceived as hues of red by humans are between 656–760 nm [53,54]. Therefore, these two hues are distinguishable by dogs and likely perceived as dark yellow/light brown/grey and blue hues [52,55,56]. In order to prevent misunderstanding regarding different color perception between humans and dogs, we will refer to the cups perceived as red by the experimenter as HR (human red) cup, and to the cups perceived as blue by the experimenter as HB (human blue) cups. Individually, the dogs were simultaneously shown a HB cup and a HR cup for 10 trials. The frequency with which they selected HR vs HB cups was designated as the color preference; if no preference was indicated, the experimenter randomly selected either HR or HB. Once color preference was decided, two HR or two HB cups were respectively placed in a left and right position on the test field. The side most frequently selected was considered the side preference, and again, if there was no difference of side selected, a side preference was randomly assigned. In addition, object preference (ball vs. jack) was evaluated for object discrimination and subsequent reversal learning tests in a similar manner. The ball was perceived as blue by the experimenter, while the jack was perceived as orange. Hues perceived as orange by humans have a wavelength of 589–656 nm [53,54], distant from the human-perceived blue wavelengths in the spectra and, therefore, distinguishable by dogs. However, because the objects used in this test had a very different shape, the latter will be considered as the primary distinctive factor in this report.

**Figure 2:**
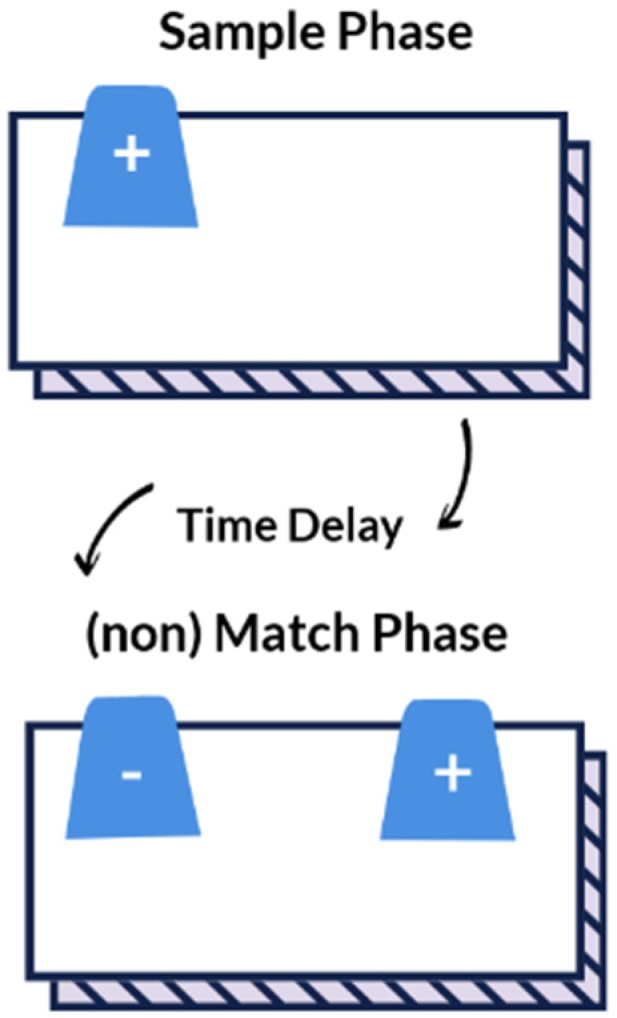
Schema of the delayed non-match to position test.

The delayed non-match to position (DNMP) task evaluates working- and visuospatial memory and has been previously described in the literature [28,29,57]. One trial consists of a sample phase, a progressive time delay interval (20-, 30-, 40-, 50-, or 60-s), followed by a non-match phase (Figure 2). For the sample phase, the dog was blinded to the test field while the experimenter placed a single plastic cup (HB or HR based on preference) upon a paper bowl to hold the food reward in the non-target location (S-) (left or right based on preference). The test field was revealed to the dog by raising the shade, which cued the dog to make a selection. For the sample phase, there was only one possible cup to select and once the dog pushed the cup with its nose, it was immediately rewarded with a dollop of canned food within the paper bowl. Afterward, the dog was again blinded to the test field by lowering the shade and the time delay was initiated. During this time, the experimenter placed two identical cups upon paper bowls at opposite sides of the test field—the target (S+) (left or right based on non-preference) and non-target (S-) location. After the set time delay passed, the shade was raised cuing the dog to make a selection. Both cups sat atop paper bowls containing a food reward, but the dog was not able to obtain the reward in the (S-) location. To prevent the dog from obtaining the food reward in the S- location, the bowl was covered by plastic wrap with holes to allow the dog to smell but not ingest the reward. Only when the dog selected the cup in the target location (S+), did the dog receive an immediate food reward within the bowl. This sequence was repeated for a total of 10 trials each session. Dogs were allowed a maximum of 80 DNMP trials (10 trials every other week for 16 weeks) for the initial DNMP task. To evaluate long-term memory, the dogs were then re-evaluated every three weeks for 27 weeks (9 sessions), and then every six weeks for 12 weeks (2 sessions). The progressively longer time intervals allowed for evaluation of recall strength. The interval assessment for post DNMP was based upon the last session completed for each dog and reported as post DNMP.

Object discrimination tests evaluated vision and working memory [43,58]. Each dog was assigned an object, positive stimulus (S+), based on their preference of either the ball or the jack. For each trial, the test field was blinded to the dog and then both objects were simultaneously shown to the dog at opposite locations to each other. The location of S+ was randomly swapped between left and right positions throughout a session. The dog was immediately rewarded when S+ was selected and received no food reward when the negative stimulus (S−) was selected. Again, both objects contained food, but the dog was not able to access the food in S− as it was securely lodged into the object, while the food reward for S+ sat immediately under a hollowed spaced within the object. Dogs were allowed a maximum of 80 trials, or once the criterion was reached, the dogs progressed to a reversal-learning test to evaluate learning flexibility.

**Figure 3:**
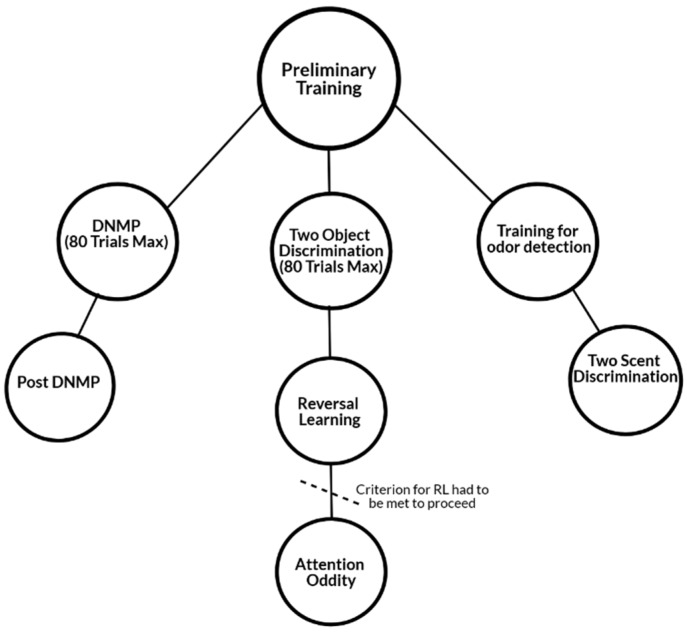
Study design flow chart showing the sequence in which cognition tests were administered. Delayed non-match to position (DNMP), visual, and scent discrimination were frequently run concurrently.

For the reversal-learning test [59,60,61], dogs were shown the same two objects used during the object discrimination test (ball vs. jack), however, the S+ and S- were reversed. Thus, the S+ from object discrimination was now the S− in reversal learning tests and vice versa. Again, the dogs were blinded to the test field and then shown the two objects simultaneously that were swapped between left and right positions. The dog was only rewarded when S+ was selected; they received no reward for selecting S−. Both objects contained a food reward, but the dog was unable to obtain food from the S− (see above). A maximum number of trials were not designated for this task. If and when dogs reached the criterion, they were moved onto an attention oddity test (Figure 3). The study was interested in both learning flexibility and attention, so it was decided to use this sequence of tasks in order to utilize familiar objects and reduce time familiarizing dogs to a new set of objects due to the anticipated disease progression of MPS I affected dogs.

**Figure 4:**
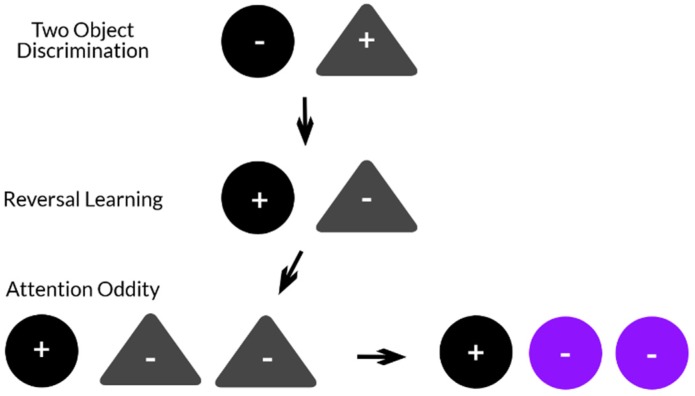
Visual stimuli and how they were used for discrimination. The same two objects were used for two object discrimination, reversal learning, and attention oddity.

The attention oddity test evaluates selective attention [41,62] and was comprised of an acquisition phase, four sessions with familiar distractors (sessions 1–4), and four sessions with non-familiar distractors (sessions 5–8) [41]. For the acquisition phase, each dog was allowed 5 sessions consisting of 12 trials per session. The attention oddity task was comprised of 8 sessions consisting of 12 trials; four trials without distractors, four trials with one distractor, and four trials with two distractors. The S+ from the reversal- learning test continued to be S+, while the S− served as familiar distractors, and non-familiar distractors differed in color (purple, as perceived by humans) and larger in size (LS, large sized) but similar in shape to the S+ (LS jack or LS ball) (Figure 4). Again the dog only received a food reward if S+ was selected (with the food reward easily accessible within the hollow space) with food lodged within but not obtainable within the S− objects. Due to their dichromatic vision, it is possible that the human-perceived colors blue and purple appear similar to a dog. Even though studies indicate that dogs will use hues to help differentiate objects in such paradigms [52,63,64], we considered size (LS) as the main discriminant factor. The number of S− (distractors) varied from zero to two. The acquisition phase consisted of 12 trials that utilized the same S+ and S− paradigm of reversal-learning task (HB ball and HO jack). This was to ensure that the dog had adequately learned which object was S+.

To evaluate olfaction and memory, dogs were trained to discriminate birch (S+). Preliminary training involved three stages—training to signal for the positive scent paired with food, signal for the positive scent without food, and finally to discriminate the positive scent from a blank (no scent) [65]. Signaling on a scent included dogs scratching at the box containing the scent or sitting in front of the selected box. The scent was placed onto a cotton tip applicator and taped inside an opaque sandwich box before subjecting it to a dog. After the criterion was reached, dogs were evaluated to determine if they could discriminate birch from anise, and birch from clove. If the dog was successful in discriminating the scent, they were immediately handed a food reward by the experimenter.

The experimental timeline detailing the age at which each dog began preliminary training, cognition testing, and if euthanized has been provided in Table 3. Dogs were assigned two testing days per week and tested on a different cognitive task per test day (i.e., test day 1: DNMP; test day 2: object discrimination). Specific cognitive tests were administered to the dogs every other week (i.e., week 1: DNMP; week 2: scent discrimination; week 3: DNMP, etcetera). The criterion was set at nine out of ten positive trials on a single test day or eight out of ten positive trials over two consecutive test days for DNMP, object discrimination, reversal learning and scent discrimination. The criterion for attention oddity was 11 out of 12 positive trials on a single day or 10 out of 12 positive trials over two consecutive test days [62,66].

For descriptive statistics, the real mean percent error and real mean number of trials to reach the criterion are reported. A mixed effects logit linear regression model was used to evaluate choice (correct versus incorrect) related to group (MPS I untreated; MPS I treated; control), age, and session × trial statistical interaction. Test sessions occurred on subsequent days, with 10 trials occurring each session. For each trial, the choice (incorrect or correct) made by the dog was recorded and used for statistical analysis. It was assumed that if the dogs were learning the task, the number of incorrect choices would improve with each session and trial. The rationale for such models is that the order of a particular session and trial may have an effect on the outcome. In order to establish the true effect of the group, the results were adjusted for the order in the experiments. Therefore the interaction of the session and trial, as well as age, were confounders. Statistical significance was assumed for a *p*-value < 0.05. A mixed effects Poisson regression was used to evaluate results of the post DNMP tests due to the non-normal distribution and limited data points. A Poisson analysis was run using choice (incorrect versus correct), group, age, time delay, and trials completed per session as the distribution was not normal, Poisson distribution. For independent variables showing complete separation (perfect prediction) in regards to the main outcome, Firth’s penalized logistic regression was used. All analysis was performed using STATA 15MP, StateCorp, State College, TX, USA.

## 3. Results

### 3.1. Subjects

Five MPS I untreated, five MPS I treated, and four MPS I heterozygotes (clinically normal) dogs were tested. Within the group of MPS I untreated dogs there were four males and one female, the MPS I treated dogs were also four males and one female, and all four of the clinically normal dogs were female. During the course of the study one MPS I treated and three MPS I untreated dogs were euthanized (see Table S2 of Supplementary Materials for additional information).

### 3.2. DNMP

A total of six dogs reached the criterion for the 20-s (s) delay (Table 4). At a 20-s delay, the MPS I untreated group committed the most errors resulting in the highest mean percent error when compared to the other groups (Table 4). Both the normal and MPS I untreated groups only had a single dog from each group reach the criterion at the 20-s delay, permitting them to move onto the 30-s time delay (Table 5). The MPS I treated group committed the least errors resulting in the lowest mean percent error, required the least number of trials to reach the criterion, and had the greatest number of dogs reach the criterion when compared to the other groups (Table 4). At the 30-s delay, the single MPS I untreated dog did not reach the criterion, while the single normal dog reached the criterion without errors (Table 5). Three of the four MPS I treated dogs reached the criterion at the 30-s time delay permitting them to move onto the 40-s time delay (Table 5). At the 40-s delay the single normal dog reached the criterion in 10 trials without errors, while only two of the three MPS I treated dogs reached the criterion (Table 6). At the 50-s time delay, the single normal dog and 2 MPS I treated dogs reached the criterion committing only a single error each (Table 7). At the start of the 60-s delay, the MPS I treated dogs had reached the 80 trial maximum and were not permitted to test at the 60-s delay. At the 60-s delay, the single normal dog was allotted 30 remaining trials but failed to reach the criterion (Table 8). Some data for groups could not be reported due to either only a single dog or no dogs reaching the specified time delay. Two MPS I treated and one normal dog did not make a choice on a trial and were not counted as either incorrect or correct. Such missing data points are accounted for via the mixed effects logistic regression model.

There was no significant difference in choice between the groups and age had no effect (Table 9). There was a statistical trend for a higher likelihood of making the correct choice for MPS-I treated versus normal dogs (Table 9).

#### Post DNMP

After completion of the DNMP test, 10 dogs were re-evaluated at time intervals previously described. All dogs were tested at a 20-s time delay unless they had reached a higher time delay, in which case, the maximum delay in which the dog reached the criterion was used. Three normal dogs (1 at 50-s, 2 at 20-s), five MPS I treated dogs (2 at 50-s, 2 at 30-s, and 1 at 20-s), and two MPS I untreated dogs (2 at 20-s) were tested. In comparison to normal dogs, MPS-I treated dogs were significantly less likely to give incorrect responses (Table 10). Time delay had a significant effect decreasing the likelihood of an incorrect answer (Table 10). There was no significant effect of age or trials completed per session.

### 3.3. Object Discrimination

All four dogs from the normal group, three dogs of the MPS I treated, and four MPS I untreated dogs met the criterion for object visual discrimination within the eight-session maximum. The normal group had the lowest mean percent error, while the MPS I untreated group had the highest mean percent error (Table 11). The average number of trials performed by all dogs for object discrimination was 51 (min 29, max 70). According to our model, there was a trend in all dogs responding with a high rate of accuracy by session 4 (40 trials). By the end of each session, most of the dogs were able to correctly identify S+. There was no significant difference in choice between the three groups and age had no significant effect (Table 12).

### 3.4. Reversal Learning

All dogs were tested for reversal learning regardless of meeting the criterion for object discrimination. Four normal dogs, four MPS I treated dogs, and one MPS I untreated dog reached the criterion for reversal learning. Due to end points as a result of disease progression four dogs were euthanized prior to completing the task, however, their trials are included in the model (* note that these dogs had participated in scent discrimination testing during this time period due to the study design). The mean percent errors to the criterion for all groups were similar; however, the normal dogs required the least number of sessions, while the MPS I untreated dogs required the most number of sessions (Table 13). All dogs failed to correctly respond in the first two trials of session 1. There was no significant difference between the three groups, though a trend between clinically normal and MPS-I untreated groups was observed (OR = 0.63, *p* = 0.090) with the MPS I untreated group less likely to choose correctly (Table 14). The average number of trials completed was 89 (min 7, max 136).

### 3.5. Attention Oddity

All four normal dogs and four MPS I treated dogs were evaluated. The one MPS I untreated dog that reached reversal learning the criterion, did not pass attention oddity acquisition phase, and therefore none of the MPS I untreated subjects were evaluated. Individual data for S+ has been shown (Table 15). However, all acquisition trials have been included in the mixed effects model (Table 16). No significant differences in age, group, or familiarity of the distractor were observed (Table 16).

### 3.6. Scent Discrimination

Two clinically normal, four MPS I treated, and three MPS I untreated dogs completed preliminary training with the mean number of trials required to pass preliminary training shown in Table 17. A firth logistic regression was used to analyze the number of preliminary training trials required to pass onto scent discrimination (Table 18). There was a significant negative effect of total number of trials the dogs experienced and the ability to complete preliminary training (*p* = 0.009) (Table 18).

One normal, four MPS I treated, and one MPS I untreated dogs were able to discriminate birch from anise and birch from clove, respectively. When preliminary training began (first introduced to birch scent), the four MPS I treated dogs were 38 weeks of age, while the normal and MPS I untreated dogs were 31 and 25 weeks of age, respectively. One MPS I untreated dog was able to discriminate between birch and anise but not between birch and clove. One normal dog passed preliminary training but was not able to reach the criterion for discrimination between birch and clove; this dog was 48 weeks when training began. One MPS I untreated dog was euthanized during this task to disease progression. There was a significant effect of age on performance with increased age having a negative impact on accuracy (Table 19). There was also a significant difference between the control, MPS-I treated, and MPS-I untreated subjects (Table 19).

## 4. Discussion

This study found several significant differences of MPS I affected dogs, however cognitive deficits were not observed. Each of the three groups were able to visually discriminate between two objects, but differences in their accuracy and ability to reach the criterion for delayed non-match to position, reversal learning, attention oddity, and scent discrimination were observed. Reaching the criterion reflected their capability to acquire and recall the contingency of a task. The duration of the research was designed to span a total of 18 months, as MPS I affected dogs begin to show clinical signs at approximately 6 months of age [39] with an expected lifespan of less than 3 years [24]. During this time, 4 MPS I affected (3 untreated and 1 treated) dogs were euthanized for progressive disease processes.

In the current study, all dogs were less than 1 year of age when DNMP testing began and less than half of the dogs reached the 20-s criterion. In previous studies of dogs performing a DNMP task, more errors were made with increasing age and dogs between 1–6 years of age perform with the highest accuracy [41,57]. One study reported an effect of age on DNMP, in which dogs less than 1 year performed similarly to middle aged dogs (>6 years of age) indicating cognitive deficits [45]. The researchers of the study hypothesized dogs less than 1 year of age may have had developmental delays, and their performance due to an immature prefrontal cortex [45]. However, for our population, age had no effect on performance; but despite no significant difference, there was a trend that MPS I treated dogs were more likely to make correct choices. This may be interpreted as a higher performing long-term memory versus different characterization of brain development of MPS I treated dogs [45]. Additionally, the longest time delay reached was 60-s by one normal dog and two MPS I treated dogs, which highlights an intact hippocampus that is required for spatial tasks [67], and working- and long-term memory. Furthermore, the post DNMP tests showed that the MPS I treated dogs were significantly less likely to give incorrect responses (*p* < 0.05). We hypothesize this may be due to inherent behavioral traits associated with MPS I versus differences in their hippocampal metabolism or development when compared to normal dogs [45]. For the post DNMP task, longer time delays significantly reduced the likelihood of an incorrect choice (*p* < 0.005); this may be a function of the increased time in which the dog had to solve the task at hand. Several studies have shown that increased time delays allow subjects more time to solve the task and improve performance [68].

Discrimination tasks are an index of learning and memory that relies on the subject to make an association between a specific stimulus (object, scent, sound), and at a future time, to recognize and recall the stimulus. Sensory discrimination sometimes referred to as a two-choice discrimination task, using olfaction, vision, or auditory capabilities require intact sensory pathways [61,67]. Both inherent differences of a stimulus or object (size, shape, color, scent, sound), natural capabilities of dogs, and experiences relevant to the dog should be considered when designing cognition tasks as previous studies suggest that certain age groups of dogs perform poorly on size discrimination [43]. The current study utilized a variety of shapes and colors to test the discrimination ability in our population of dogs. However, there was no significant difference among the three groups in their ability to discriminate between two objects differing in shape and color. Object discrimination is considered a simple task, requiring an intact rhinal cortex [67], not an intact hippocampus, which highlights that the dogs reaching the criterion had an intact rhinal cortex. Several reasons including individual differences in rate of learning or rhinal cortex dysfunction may explain why three dogs did not reach the criterion (one MPS untreated and two MPS treated dogs). Allowing for additional test sessions may help differentiate between rates of learning versus rhinal cortex dysfunction. The majority of dogs reached the criterion in four sessions or by the 40th trial, exceeding our premise that it would require dogs’ 80 trials to learn the contingency.

After the maximum of 80 trials for object discrimination, each of the 14 dogs was started on a reversal-learning task (regardless of reaching the criterion). This task investigates learning flexibility; an executive function that requires subjects to inhibit previously learned associations. Executive function requires an intact prefrontal cortex and is responsible for simultaneously coordinating several cognitive domains to produce a higher order response [44]. Reversal learning has been shown to be a sensitive tool for disease progression in neurodegenerative disease [61]. In our study, there was no significant difference among the three groups in their reversal learning ability. However, there was a trend of the MPS I untreated group being less likely to make a correct choice. These results may be confounded due to four MPS I affected dogs (three untreated and one treated) being euthanized and therefore not completing the test. Furthermore, the MPS I untreated dogs had the highest number of mean sessions compared to MPS I treated and normal dogs for the criterion. Reversal learning studies in dogs report reduced learning flexibility with increasing age, as a higher number of errors in aged dogs may reflect a higher level of perseverance [59,61,69]. Therefore, our data may suggest that MPS I untreated dogs exhibit prefrontal cortex dysfunction as characterized by their reduced ability to inhibit previously learned associations. We also attribute each of the 14 dogs’ incorrect choices on the first two trials to be due to perseverance of their previous learned association and long term memory retention [69,70].

Another aspect of inhibitory control is the ability to selectively attend to relevant information in the environment [43,44]. Attention has been described as having five components: focused, sustained, selective, alternating, and divided [71]. The ability to filter and attend to relevant information in an individual’s surroundings is referred to as selective attention [72]. Attention is involved in many aspects of cognition and memory, and in humans is gradually developed and refined from infancy to adulthood [73]. Attention oddity tasks have been used to evaluate selective attention in both humans and dogs [62,73,74,75]. In the study presented, only dogs that reached the criterion for reversal learning were tested on an attention oddity task. The attention oddity task was one of the last cognitive tests performed, and by this test period, the dogs were just over 1 year of age and several had been euthanized due to disease progression. Only a single MPS I untreated dog reached the criterion for reversal learning moving onto the attention oddity task, however, the dog did not pass the acquisition phase. This was unexpected as the contingency and objects used for the acquisition phase was the same as the reversal-learning task. This dog was 68 weeks old when this cognitive task began, and his failure may have been due to an attention deficit, a degradation of long-term memory, or a decline in vision secondary to corneal clouding [39]. A higher number of MPS I untreated dogs would be required to fully investigate such possibilities. Age had no effect on the outcome of this cognitive task and no statistical significance was observed in either the MPS I treated or MPS I untreated dogs compared to the normal dogs. These results may be due to the age of our population as a study of 145 pet Border collies (6 months to 14 years of age), found that selective attention peaks at the age of 3–6 years [75]. Our study found no significant effect on performance when the distractors used were familiar or unfamiliar. However, these results differ from a study investigating selective attention in beagles (mean age of 11.6 years), which reported a higher number of errors occurred when the number of distractors increased regardless of memory performance [41]. It is important to point out that there is a difference in age between our population of dogs and the aged population of beagles in the latter study.

A scent discrimination task requires a subject to differentiate between at least two different odors and requires an intact olfactory system, pyriform-, entorhinal-, and orbitofrontal cortices [76]. Prior to the dogs discriminating between two scents, they were required to pass an acquisition phase. Only half of the normal group completed preliminary training, while a majority of the MPS I affected dogs passed. A firth logit regression model for the preliminary training trials showed that the more trials a dog experienced, the less likely they were to pass preliminary training. This may reflect inherent differences of individual dogs [65] and not necessarily due to pathology. A previous study of pet dogs, various ages (1–11 years of age), have successfully been trained to discriminate odor though not every pet dog was able to perform above chance [65].

The MPS I affected groups, treated and untreated, were more likely to correctly discriminate between two scents when compared to the normal group. For this cognitive task, age had a significant effect on performance for each of our groups. Two of the clinically normal dogs, 31 weeks and 48 weeks of age when first exposed to the birch scent during scent discrimination training, passed training. However, the clinically normal dog that began training at 48-weeks failed to discriminate between birch and anise and therefore was not tested with clove. All but one of the MPS I treated dogs, all 38 weeks of age when scent discrimination training began, passed and were able to discriminate between birch and anise, as well as between birch and clove. Though three MPS I untreated dogs passed scent discrimination training, one was euthanized prior to completing the birch versus anise discrimination test. The other two MPS I untreated dogs, began scent discrimination training and therefore exposed to the birch scent at 25 weeks of age; these dogs were able to discriminate birch from anise and birch from clove. This was an unexpected finding, though no previous olfaction studies in MPS I affected dogs exist, a study of MPS I affected cats showed that their olfactory epithelia were structurally disorganized and that olfactory receptors were less likely to respond to odors [77]. Given this fact, we would have expected that MPS I affected dogs are less likely to correctly discriminate scents when compared to normal dogs. It is known that age related changes of the olfactory system in dogs occurs at 14 years of age and older [78] but when compared to humans, little information exists on other factors that may influence early changes in a dog’s olfactory system, such as disease. Based on these results, we speculate that the expected changes in olfaction in MPS I affected dogs did not occur in our dogs due to the protective effect of early training on the epithelium. The progressive declines in olfaction of MPS I affected dogs as they age may be further elucidated by training MPS I affected dogs at an age older than 25 weeks or carrying on olfaction testing past 81 weeks of age. Further investigation is worthwhile, as impaired olfaction can be associated with declines in cognition [79], though hyposmia and anosmia is not noted in humans affected with MPS I nor fully investigated in MPS I dogs.

Differences in the cognitive ability of MPS I treated and untreated dogs were observed for several tasks, though not all reached statistical significance. The MPS I treated group were significantly more likely to correctly respond on the post DNMP task when compared to the normal and MPS I untreated groups. Both the MPS I untreated and treated groups were significantly more likely to correctly discriminate between two scents when compared to the normal group. These results indicate that our population of MPS I treated dogs have a superior long term spatial memory than either normal or MPS I untreated groups. We also report that MPS I affected dogs have an intact olfactory system; the MPS I untreated and treated dogs had a superior ability to focus on and/or detect olfactory stimuli than normal dogs. No significant differences were observed for spatial working memory, attention, or executive function. This study did not find any significant deficits between the normal and MPS I untreated group. However, it should not be disregarded that three MPS I affected dogs (two treated and one untreated) failed to reach the criterion for object discrimination and only one MPS I untreated dog reached the criterion for reversal learning in comparison to the normal group. Failure to reach the criterion for object discrimination may be an indicator of disease progression, as out of the three MPS I affected dogs that failed, two (one treated and one untreated) were later euthanized during reversal learning testing. Lastly, vision loss secondary to corneal clouding may have contributed to findings for cognition tests run at a later period, such as the last half of reversal learning and attention oddity.

Failure to find differences between the normal and MPS I untreated groups may be due to sex bias of the normal group (all female), the age at which testing began, the small sample utilized, or a combination of all such factors. The clinically normal dogs also cannot be compared to companion dogs due to the different socialization and experiences they have been exposed to [43,80]. The experimenter noted that normal appearing dogs exhibited signs of stress and anxiety, such as pacing, scanning of the environment, panting, and dilated pupils. This behavior was in contrast to the non-normal appearing dogs that remained near the apparatus and focused on the experimenter. However, stress and anxiety levels were not quantitatively measured. Despite best efforts and enrichment provided to laboratory kept dogs, studies indicate they are likely to experience chronic or situational stress due their environment [81,82]. In these settings, female dogs may exhibit stronger acute stress responses than males [81,83], which further supports previous findings that female dogs tend to suffer from anxieties more frequently than male dogs [84,85]. Studies investigating the role of sex hormones on the stress response in humans and rats highlight the effects of estrogen levels and activation of different brain regions in males and females [86,87]. These studies suggest high levels of estrogen promote prefrontal cortex dysfunction [86,88]. Additionally, chronic stress in rats has been shown to reduce learning and memory [89] as well as induce damage to the prefrontal cortex and hippocampus [90]. We speculate that the anecdotal behavioral differences between MPS I affected and normal dogs (in laboratory settings) may be attributed to neuroarchitectural differences within the central nervous system, specifically the prefrontal cortex and hippocampus. Further studies to describe the relationship between hormones and CNS activation during times of stress in dogs are greatly needed. Further needed are social cognition studies of MPS I affected dogs, both laboratory and household pets, to confirm these subjective observations.

We found that performing cognitive tests in MPS I affected dogs was feasible. We describe testing procedures and an apparatus that proved successful for testing dogs with physical changes that occur with MPS I and suggest improvements to refine several cognitive protocols. For the post DNMP, we found that dogs were significantly more likely to respond correctly with longer time delays. This may be an artifact of learning as dogs at longer time delays, were successful at passing the initial criterion. However, future studies may prefer to start at a longer time delay to determine if the additional time would benefit the dogs’ ability to process the task. For preliminary training of scent discrimination, dogs were significantly less likely to pass the training phase the more trials it underwent. This knowledge may support having a maximum number of training trials to not only exclude dogs’ less likely to discriminate, but also reduce time and materials spent on training. We also recommended an expanded scent discrimination task following aged MPS I dogs to determine if their ability declines as our MPS I population at the time of scent discrimination training was less than 1 year. Additionally, we recommend a reversal-learning task separate from an attention oddity task, as well as starting attention oddity at a younger age in order to better determine if vision deficits contributed to our current study. As shown by the results presented on olfaction discrimination, it is important to remind dog owners that exposing dogs to an enriched environment at a very early age may be helpful in minimizing cognition changes secondary to disease.

Though these results provide a first evidence of differences in cognitive ability in dogs affected with MPS I, as well as a foundation for further research in the field, there are limitations to the current study, including the sample size that may impact significance [91]. Clinical cases of dogs with naturally occurring MPS I are extremely rare and, even if the disease can be reproduced in laboratory dogs, the availability of the latter is still limited [34]. US federal, state, local, and institutional laws and regulations impose high standards and restrictions for the use of dogs as laboratory animals. These standards are set to guarantee a continued effort to reduce the use of canine subjects and to refine experimental protocols. Pilot studies with small samples are therefore used to assess the viability of research protocols.

Another potential limitation may be represented by the different color perception between humans and dogs, which makes somewhat complex understanding how dogs see the different objects used for cognitive testing. Dogs do not see colors the same way humans do, for example they do not see objects that look red to us as so. However, they are able to differentiate one color or hue from another, unless both of the colors/hues are close to the neutral point of 480 nm in the spectra of absorption of canine cones [52,55]. As previously explained, dogs can differentiate human-perceived blue (HB) from human-perceived red (HR) or orange (HR), but may find more difficult differentiating human-perceived blue (HB) from human-perceived purple (HP) because both hues are closer to the neutral point. The color of an object was used as the only discriminant factor only in the initial color preference test, in which case we used two cups with colors (HB and HR) corresponding to distant wavelengths on the spectra of absorption. For all the other tests, the color of the object was never the only factor that dog could use for discrimination, making this factor much irrelevant in evaluating the choice of a tested dog.

Using laboratory animals for rare diseases naturally occurring in dogs is necessary to investigate medical, behavioral, and cognitive changes under standardized conditions [34] but represents a limitation at the same time. The dogs used in this study were laboratory bred and kept. Being laboratory dogs influences their environmental exposure and experiences, as well as their coping mechanisms in stressful circumstances [80]. Therefore, extrapolations between laboratory and companion dogs need to be carefully considered. Being dogs with MPS I an accepted model of MPS I in humans [35,92], this study may also serve as framework for studying cognitive effects of other medical conditions common to dogs and humans.

The cognitive and behavioral effects of many medical diseases have not been fully investigated despite behavior changes (such as loss of appetite, reduce interaction, etc.) being the first sign of ailment in companion animals. This research highlights the need for veterinarians to collect baseline information of a pet’s cognitive ability and its evolution so that the clinician can be sensitive to changes if they occur. If cognition tests cannot be performed in such a setting [93], a string of history questions can be discussed with the owner, as cognitive changes can occur early from chronic inflammation [94], as opposed to being a primary sequela of the disease. Some may feel overwhelmed by the intense training involved with cognition tests; however, studies have shown the possibility of performing cognition testing in a clinical setting may be helpful in assessing cognitive decline in pets with chronic medical conditions in order to provide appropriate interventions, including additional diagnostics, diet changes, or enrichment [94,95].

## 5. Conclusions

This pilot study has demonstrated that cognition testing can be successfully performed with MPS I affected dogs. The MPS I treated group significantly outperformed the normal group on the post-DNMP task, suggesting a superior long term and spatial memory. This was not surprising as more MPS I treated dogs reached the DNMP criterion than normal dogs, indicating that the normal dogs failed to learn the contingency of the test despite additional trials, while the MPS I treated dogs recalled the contingency despite increasing time intervals. Additionally, the MPS I treated and untreated dogs had superior olfaction abilities to discriminate birch when compared to normal dogs. This may be explained to an age effect when first exposed to the scent, in which increased age was found to have a negative effect on accuracy. No significant differences were observed for working memory, attention, or executive function. The cognitive changes described demonstrate that MPS I influences dog cognition and highlights the potential that other storage diseases may affect cognition in dogs as they do in humans. Future neurocognitive investigations of medical disorders can provide knowledge that may help to improve quality of life and comprehensively assess future interventions.

Considering the limited availability of dogs with MPS I in clinics and the lab, together with their short life expectancy, we recommend that future studies focus on a specific cognitive domain in order to better adapt specific protocols to the expected evolution of the domain being tested. For example, olfaction-testing protocols may be developed to detect the potential effect of early training at different ages. Additionally, object discrimination tests can be designed such that the number of trials administered is 40 instead of 80. From a clinical aspect, these findings stress the importance of inquiring about a dog’s behavior (focus, attention, ability to discriminate sensory stimuli), which may be beneficial in detecting cognitive changes and an opportunity to improve welfare.

## Figures and Tables

**Table 1:** Demographics of study population. Identification number, mucopolysaccharidosis I (MPS I) status, clinical status, and sex (all dogs intact).

ID #	MPS I Status	Clinical Status	Sex
I-722	Carrier	Normal	F
I-670	Carrier	Normal	F
I-682	Carrier	Normal	F
I-703	Carrier	Normal	F
I-683	Affected	Treated	F
I-687	Affected	Treated	M
I-690	Affected	Treated	M
I-691	Affected	Treated	M
I-692	Affected	Treated	M
I-672	Affected	Untreated	M
I-680	Affected	Untreated	M
I-720	Affected	Untreated	F
I-725	Affected	Untreated	M
I-726	Affected	Untreated	M

**Table 2:** Group preference evaluation results. Data presented for the selected preference of each individual for color, object, and side. HB = human perceived blue; HR = human perceived red.

ID #	Sex	Color	Side	Object
Group 1: Clinically normal				
I-670	F	HB	Right	Jack
I-682	F	HR *	Left	Ball *
I-703	F	HB	Left *	Ball
I-722	F	HB	Left	Ball
Group 2: MPS I Affected Treated				
I-683	F	HB	Right	Ball
I-687	M	HR *	Left	Ball
I-690	M	HR	Right	Ball
I-691	M	HB	Right	Jack
I-692	M	HB	Right	Jack
Group 3: MPS I Affected Untreated				
I-672	M	HR	Left	Ball
I-680	M	HR *	Left	Ball
I-720	F	HB	Left	Ball
I-725	M	HR	Right	Ball
I-726	M	HR	Left	Jack *

* Randomly assigned due to lack of preference.

**Table 3:** Experimental timeline: age of dogs for which training, individual cognitive task, and euthanasia was initiated.

Age (weeks)								
ID #	Preliminary Training	DNMP	Object Discrimination	Reversal Learning	Attention Oddity	Scent Discrimination Training	Scent Discrimination	Euthanasia for Disease Progression
Group 1: Clinically normal (MPS I Carriers, Heterozygote)								
I-670	19	31	33	47	63	48	68	
I-682	11	24	26	34	52	39	CNM	
I-703	9	20	22	28	68	36	CNM	
I-722	8	36	35	41	65	31	48	
Group 2: MPS I Affected Treated								
I-683	11	24	26	36	54	38	81	
I-687	11	24	26	34	72	38	68	
I-690	11	24	26	42^E^	Euthanized	38	CNM	68
I-691	11	24	26	42	61	38	64	
I-692	11	24	28	42	75	38	78	
Group 3: MPS I Affected Untreated								
I-672	19	31	36	47 ^E^	Euthanized	46^E^	Euthanized	52
I-680	12	25	27	37	68	40	CNM	
I-720	8	29	26	42	CNM	25	49	
I-725	8	29	26	40 ^E^	Euthanized	25	44	57
726	8	47	43	59 ^E^	Euthanized	31	56 ^E^	60

CNM = criterion not met. ^E^ = euthanized during testing period.

**Table 4:** DNMP descriptive statistics by group for 20-s delay. This includes the number of dogs tested (N) from each group, mean (real) percent error of each group, mean (real) number of trials performed by each group, 95% confidence interval, standard deviation, and number of dogs from each group that reached the criterion at the 20-s delay.

Group	N	Mean Percent Error	Mean Number of Trials	Upper 95% CI	Lower 95% CI	SD	Number of Dogs that Reached Criterion
**Normal**	4	0.639	63.75	1	0.176	0.291	1
**MPS I untreated**	5	0.751	69.00	1	0.499	0.203	1
**MPS I treated**	5	0.506	49.4	0.684	0.328	0.143	4

**Table 5:** DNMP descriptive statistics by group for 30-s delay. This includes the number of dogs tested (N) from each group, mean (real) percent error of each group, mean (real) number of trials performed by each group, 95% confidence interval, standard deviation, and number of dogs from each group that reached the criterion at the 30-s delay.

Group	N	Mean Percent Error	Mean Number of Trials	Upper 95% CI	Lower 95% CI	SD	Number of Dogs that Reached Criterion
**Normal**	1	0	10	---	---	---	1
**MPS I untreated**	1	0.367	30	---	---	---	0
**MPS I treated**	4	0.100	15	0.212	0	0.071	3

**Table 6:** DNMP descriptive statistics by group for 40-s delay. This includes the number of dogs tested (N) from each group, mean (real) percent error of each group, mean (real) number of trials performed by each group, 95% confidence interval, standard deviation, and number of dogs from each group that reached the criterion at the 40-s delay.

Group	N	Mean Percent Error	Mean Number of Trials	Upper 95% CI	Lower 95% CI	SD	Number of Dogs that Reached Criterion
**Normal**	1	0	10	-----	----	-----	1
**MPS I treated**	3	0.067	9.667	0.210	0	0.058	2

**Table 7:** DNMP descriptive statistics by group for 50-s delay. This includes the number of dogs tested (N) from each group, mean (real) percent error of each group, mean (real) number of trials performed by each group, 95% confidence interval, standard deviation, and number of dogs from each group that reached the criterion at the 50-s delay.

Group	N	Mean Percent error	Mean Number of Trials	Upper 95% CI	Lower 95% CI	SD	Number of Dogs that Reached Criterion
**Normal**	1	0.100	10	---	---	---	1
**MPS I treated**	2	0.050	10	---	---	0.071	2

**Table 8:** DNMP descriptive statistics by group for 60-s delay. This includes the number of dogs tested (N) from each group, mean (real) percent error of each group, mean (real) number of trials performed by each group, 95% confidence interval, standard deviation, and number of dogs from each group that reached the criterion at the 60-s delay.

Group	N	Mean Percent Error	Mean Number of Trials	Upper 95% CI	Lower 95% CI	SD	Number of Dogs that Reached Criterion
**Normal**	1	0.5	30	---	---	---	0
**MPS I treated**	2 *	-----	-----	-----	-----	-----	0

* These dogs successfully reached the criterion at the 50-s delay but had reached the 80 trial maximum and, therefore, not eligible to be tested at the 60-s delay.

**Table 9:** DNMP mixed effects logistic regression model. Analysis of MPS I affected groups compared to normal group for choice (correct versus incorrect) at each session x trial and age.

Predictor	Odds Ratio	Robust Std Error	Z	P	95% CI
**Age**	1.0047	0.0139	0.34	0.735	0.9778–1.0322
**MPS I treated**	8.5858	10.0060	1.84	0.065	0.8745–84.2909
**MPS I untreated**	0.4006	0.5479	−0.67	0.504	0.0274–5.8455
**Time delay**	1.0329	0.5569	0.60	0.549	0.9292–1.1480

Note: STATA programming code: melogit choice0incorrect1correct age i.session#i.trial i.group timedelays i.reachedcriterion0no1yes || subject: > or vce (robust).

**Table 10:** Post DNMP mixed effects Poisson regression model. Analysis of MPS I affected groups compared to normal group for choice (correct versus incorrect) at each session x trial, age, time delay, and trials completed.

Predictor	IRR	Robust Std Error	Z	P	95% CI
**Age**	1.0042	0.0028	1.52	0.129	0.9988–1.0097
**MPS I treated**	0.5681	0.1433	−2.24	0.025	0.3465–0.9313
**MPS I untreated**	0.9523	0.1489	−0.31	0.756	0.7013–1.2939
**Time delay**	0.9717	0.0092	−3.04	0.002	0.9539–0.9899
**Trials completed per session**	1.7156	2.6530	0.35	0.727	0.0828–35.5376

Note: STATA programming code: mepoisson ofincorrectresponses i.group age timedelays i.trialscompleted#i.session || id: irr vce (r > obust).

**Table 11:** Two object visual discrimination descriptive statistics for each group. This includes the number of dogs tested (N) from each group, mean (real) percent error of each group, mean (real) number of sessions performed by each group, and number of dogs from each group that reached the criterion in the allotted 80 trial maximum.

Group	N	Mean Percent Error	Mean Sessions to Criterion	Number of Dogs Reached Criterion
**Normal**	4	0.153	4.25	4
**MPS I untreated**	5	0.208	6.5	4
**MPS I treated**	5	0.198	5.3	3

**Table 12:** Object discrimination mixed effects logistic regression model. Analysis of MPS I affected groups compared to normal group for choice (correct versus incorrect) at each session x trial and age.

Predictor	Odds Ratio	Robust Std Error	Z	P	95% CI
**Age**	1.0001	0.0022	0.06	0.954	0.9959–1.0044
**MPS I treated**	0.9612	0.3142	−0.12	0.904	0.5065–1.8241
**MPS I untreated**	0.7153	0.2086	−1.15	0.251	0.4038–1.2669

Note: STATA programming code: melogit choice0incorrect1correct age i.session#i.trial i.group || id: or vce (robust).

**Table 13:** Reversal learning descriptive statistics for each group. This includes the number of dogs tested (N) from each group, mean (real) percent error of each group, mean (real) number of sessions performed by each group, and number of dogs from each group that reached the criterion.

Group	N	Mean Percent Error	Mean Sessions to Criterion	Number of Dogs Reached Criterion
**Normal**	4	0.580	12.25	4
**MPS I untreated**	5	0.590	16	1
**MPS I treated**	5	0.544	13.25	4

**Table 14:** Reversal learning mixed effects logistic regression model. Analysis of MPS I affected groups compared to normal group for choice (correct vs. incorrect) at each session x trial and age.

Predictor	Odds Ratio	Robust Std Error	Z	P	95% CI
**Age**	1.0035	0.0034	1.03	0.303	0.9968–1.0103
**MPS I treated**	0.8429	0.2477	−0.58	0.561	0.4739–1.4995
**MPS I untreated**	0.6284	0.1723	−1.69	0.090	0.3671–1.0755

Note: STATA programming code: melogit choice0incorrect1correct age i.session#i.trial i.group || id: or vce (robust).

**Table 15:** Individual data of attention oddity S+. Dog identification, group, age testing began for attention, S+, and familiar, non-familiar distractors (objects) used. LS = large sized.

ID	Group	Age Testing Began (week)	S+	Familiar Distractor	Non-Familiar Distractor
**I-670**	Normal	63	ball	jack	LS ball
**I-703**	Normal	68	jack	ball	LS jack
**I-722**	Normal	65	jack	ball	LS jack
**I-682**	Normal	52	jack	ball	LS jack
**I-691**	MPS I treated	61	ball	jack	LS ball
**I-683**	MPS I treated	54	jack	ball	LS jack
**I-687**	MPS I treated	72	jack	ball	LS jack
**I-692**	MPS I treated	75	ball	jack	LS ball
**I-680**	MPS I untreated	68	jack	ball	Not tested

**Table 16:** Attention oddity mixed effects logistic regression model. Analysis of MPS I affected groups compared to normal group for choice (correct versus incorrect) at each session x trial and age.

Predictor	Odds Ratio	Robust Std Error	z	p	95% CI
**Age**	0.9987	0.0061	−0.21	0.830	0.9867–1.0108
**MPS I treated**	1.0416	0.4703	0.09	0.928	0.4299–2.5237
**MPS I untreated**	0.2232	0.2113	−1.58	0.113	0.349–1.4277
**Familiar object vs. unfamiliar object**	2.41 × 10^−8^	0.0000	−0.00	0.996	----

Note: STATA programming code: melogit choice0incorrect1correct age i.session2#i.trial i.group1normal2affectedtrea i.familiarobject0no1yes.

**Table 17:** Preliminary training for scent discrimination by group. This includes the number of dogs tested (N) from each group, mean (real) number of trials completed to pass training, and the number of dogs that successfully completed preliminary training from each group.

Group	N	Mean Trials Completed to Pass Preliminary Training	Number of Dogs Passed Preliminary Training
**Normal**	4	131	2
**MPS I untreated**	5	155	3
**MPS I treated**	5	185	4

**Table 18:** Preliminary training for scent discrimination firth logistic regression model. Analysis of MPS I affected groups compared to normal group for choice (correct versus incorrect) at each session x trial for preliminary training.

Predictor	Coefficient	Std Error	Z	P	95% CI
**Total trials**	−0.0303	0.0115	−2.63	0.009	−0.0530–−0.0077
**MPS I treated**	3.8318	2.6604	1.44	0.150	−1.3826–9.0462
**MPS I untreated**	−0.2776	2.0656	−0.13	0.893	−4.3262–3.7710

Note: STATA programming code: xi: firthlogit outcome2 totaltrials i.group.

**Table 19:** Scent discrimination mixed effects logistic regression model. Analysis of MPS I affected groups compared to normal group for choice (correct versus incorrect) at each session x trial and age.

Predictor	Odds Ratio	Robust Std Error	Z	P	95% CI
**Age**	0.9997	0.0001	−4.75	0.000	0.9996–0.9998
**MPS I treated**	9.6068	6.1952	3.51	0.000	2.7143–34.0019
**MPS I untreated**	4.7610	2.3158	3.21	0.001	1.8351–12.3517

Note: STATA programming code: melogit choice0incorrect1correct age i.session2#i.trial i.group || id: or vce (robust).

## Supplementary Materials

The following are available online at https://www.mdpi.com/2076-2615/10/3/397/s1, Table S1: Comparison of clinical signs and behavior changes associated with lysosomal storage diseases in dogs and humans [96,97,98,99,100,101,102,103,104,105,106,107,108,109,110,111,112,113,114,115,116,117,118,119,120,121,122,123], Table S2: Causes for euthanasia of dogs during study.
