# Supplementary material for: Cognitive Abilities of Dogs with Mucopolysaccharidosis I: Learning and Memory
Source: Animals (Basel). 2020 Feb 28;10(3):397. doi: 10.3390/ani10030397 (PMC7143070; doi:10.3390/ani10030397)
Supplement: Supplementary file 1 [file animals-10-00397-s001.zip › MPS Article_Supplementary Material.docx]

**Supplementary Materials**

Table S1: Comparison of clinical signs and behavior changes associated with lysosomal storage diseases in dogs and humans.

| **Disease** | **Deficient Enzyme / Major organs involved** | **Clinical signs** | | **Behavior changes** | |
| --- | --- | --- | --- | --- | --- |
|  |  | **Dogs** | **Humans^97,98^** | **Dogs** | **Humans^97,98^** |
| Mucopolysaccharidosis I ^96^*  (Hurler, Scheie, and Hurler-Scheie) | Enzyme: alpha-L-iduronidase  Major organs involved: CNS, connective tissue, heart, skeleton, cornea | Stunted growth, facial dysmorphia, thick chin tissue, droopy eyelids, corneal clouding, umbilical hernias, lameness, joint laxity, degenerative osteoarthritis, spinal cord compression.  *Age of onset*: 8-12 weeks  *Life span:* 2-3 years | Macrocephaly, macroglossia, large lips, cheeks, nose, and vocal cords (deep voice), sleep apnea, hydrocephalus, hepatosplenomegaly, umbilical and inguinal hernias, deafness, corneal clouding, carpal tunnel syndrome, spinal stenosis, heart valve abnormalities, short stature, joint deformities, dysostosis multiplex, frequent upper respiratory and ear infections.^99^  *Age of onset*: 1 to 10 years (depending on severity)  *Lifespan*: 10 years to early adulthood (severe to attenuated) | Not documented | Developmental delays and regression. |
| Mucopolysaccharidosis II (Hunter) ^96^* | Enzyme: Iduronate 2-sulfatase  Organs involved: CNS, connective tissue, heart, skeleton | Facial dysmorphia, thick toes, ataxia, exercise intolerance, osteopenia, and degenerative neurological disease.  *Age of onset*: unknown  *Lifespan*: unknown | Macrocephaly, hydrocephalus, hepatosplenomegaly, umbilical and inguinal hernias, deafness, joint deformities, and heart valve abnormalities.^99^  *Age of onset*: 2-4 years  *Lifespan:* 10-20 years | Not documented | Hyperactivity, attention deficits, difficulty following directions, aggression, and inability to sense danger. |
| Mucopolysaccharidosis IIIA (Sanfilippo Type A) ^96^* | Enzyme: Heparan sulfamidase  Organs involved: CNS | Ataxia, hypermetria, falling and stumbling, postural deficits, and difficulty jumping.  *Age of onset:* 1.5 - 3 years  *Lifespan*: 1.5 to 7 years | Dense calvaria, coarse facial features and hair, asymmetric septal hypertrophy, seizures, deafness, loss of vision, and inability to sleep for more than a few hours at a time.^99^  *Age of onset*: 2-5 years  *Lifespan*: 20 years | Loss of learned behaviors.^100^ | Progressive dementia, aggressive behavior, and hyperactivity. |
| Mucopolysaccharidosis IIIB (Sanfilippo Type B) ^96^* | Enzyme: N-acetyl-alpha-d-glucosaminidase  Major organs involved: CNS | Tremors, stumbling, ataxia, wide base stance, corneal dystrophy, and retinal degeneration.  *Age of onset*: 2 years  *Lifespan*: 5 years | Coarse facial features, cardiomegaly, asymmetric septal hypertrophy, seizures, deafness, loss of vision, and inability to sleep for more than a few hours at a time.^99^  *Age of onset*: 2-5 years  *Lifespan*: Teenage years | Not documented | Progressive dementia, aggressive behavior, and hyperactivity. |
| Mucopolysaccharidosis VI^96^*  (Maroteaux-Lamy) | Enzyme: arylsulfatase B  Major organs involved: Skeleton, cornea, heart | Facial dysmorphia, prognathism, blunt facial features, and stunted growth.  *Age of onset*: 4-8 weeks  *Lifespan*: 1-3 years | Skeletal dysplasia, short stature, dysostosis multiplex, degenerative joint disease, cervical cord compression, meningeal thickening, bony stenosis, macrocephaly, hydrocephalus, macroglossia, hepatosplenomegaly, umbilical and inguinal hernias, heart valve abnormalities, optic nerve atrophy and blindness.^99^  *Age of onset*:  Infancy or childhood  *Lifespan*: 20 to 50 years (early to late onset) | Not documented | No intellectual deficits appreciated. |
| Mucopolysaccharidosis VII (Sly) ^96^* | Enzyme: beta-D-glucuronidase  Major organs involved: CNS, connective tissue, skeleton, heart | Stunted growth, facial dysmorphia, low set droopy ears, corneal clouding, prognathism, umbilical hernias, thickened paws, hunched gait, joint effusion, hip and patellar luxation, and loss of hind limb use.  *Age of onset*: 4 - 6 weeks  *Lifespan*: 6m – 2 years | Neonatal non-immune hydrops fetalis, coarse facial features, macroglossia, hepatosplenomegaly, corneal clouding, umbilical and inguinal hernias, small stature, severe hypotonia, neurological involvement, thoracic kyphosis, scoliosis, dysostosis multiplex, hip dysplasia, joint contracture, decreased pulmonary function, corneal clouding, and cardiac valve abnormalities.^99^  *Age of onset:* Neonatal to early childhood  *Lifespan*: birth to adulthood (dependent on severity) | Not documented | Developmental delays and progressive intellectual disability (mental retardation). |
| beta-Mannosidosis | Enzyme: beta -mannosidase  Major organs involved: CNS, skeleton, liver, spleen | Failure to grow, stiff, deafness, seizures, ataxia, and proprioceptive deficits.^101^  *Age of onset*: 8-10 weeks^101^  *Life span:* 8 months^101^ | Prominent foreheads, large ears and tongue, thick lips, communicating hydrocephalus (secondary headaches, vomiting, and vision problems), seizures, deafness, learning difficulties, joint stiffness, and frequent upper respiratory infections.  *Age of onset:* Infancy to adolescence  *Life span:* depends on severity of disease, early to late adulthood | Not documented | Intellectual disability, delayed motor development, introverted, depression, hyperactivity, impulsivity, and aggression. |
| Fucosidosis | Enzyme: alpha-L-fucosidase  Major organs involved: CNS, spleen, musculoskeletal, liver | Ataxia, dysphonia, dysphagia, hearing/visual deficits, weight loss, and seizures.^102^  *Age of onset:* 6-24 months^102^  *Lifespan:* 4 years^102^ | Impaired growth, dysostosis multiplex, seizures, abnormal muscle stiffness, angiokeratomas, distinctive facial features, recurrent respiratory infections, and visceromegaly.  *Age of onset*: Infancy to 2 years  *Lifespan*: 10 to 20 years | Progressive cognitive dysfunction, anxiety, and personality changes.^102^ | Progressive motor and cognitive impairments. |
| Globoid cell leukodystrophy (Krabbe disease) | Enzyme: galactocerebrosidase  Major organs: CNS | Ataxia, leg crossing, head tremors, paresis, poor weight gain, vision deficits, urinary incontinence, and muscle weakness^103^  *Age of onset*: 4-6 weeks^103^  *Lifespan*: 3-5 months^103^ | Developmental delays, seizures, limb stiffness, optic atrophy, hearing and vision deficits, muscle spasticity, ataxia, and progressive psychomotor decline. *Age of onset:* infancy *Lifespan*: 2 years | Dementia.^104^ | Extreme irritability. |
| Glucocerebrosidosis  (Gaucher disease) | Enzyme: beta-glucocerebrosidase  Major organs involved: CNS, spleen, liver, bone marrow | Incoordination and tremors.^105^  *Age of onset:* 4-6 months^105^  *Lifespan:* 8 months^105^ | *Non-neuronopathic form:* hepatosplenomegaly and skeletal deformities. *Infantile* *Neuronopathic form*: anemia and hepatosplenomegaly.  *Age of onset:* 4-5 months  *Lifespan*: 2 years  *Juvenile neuronopathic form:* hepatosplenomegaly, ataxia, myoclonic seizures, and spasticity.  *Age of onset:* late childhood  *Lifespan*: adulthood | Not documented | Cognitive deficits. |
| Glycogen storage disease II (Pompe) | Enzyme: alpha-glucosidase  Major organs involved: CNS, muscle, heart | Coughing, muscle weakness, panting, delayed growth, cardiomyopathy, and megaesophagus.^106,107^  *Age of onset*: 7 months^106,107^  *Lifespan*: by 2 years^106,107^ | *Classic infantile form*: progressive muscle weakness/hypotonia, cardiomyopathy, failure to thrive, and respiratory insufficiency.  *Age of onset*: < 12 month  *Lifespan*: 1 year  *Late-onset form*: progressive muscle weakness and breathing problems.  *Age of onset*: < or > 12 months  *Lifespan*: adulthood | Not documented | Cognitive delays |
| GM 1 Gangliosidosis 1 | Enzyme: beta-galactosidase  Major organs: CNS, skeleton, viscera | Vision deficits, lethargy, and difficulty walking.^108^  *Age of onset*: 2 - 4 months^108^  *Lifespan*: 8 – 15 months^108^ | Developmental delays, muscle weakness, hepatosplenomegaly, skeletal abnormalities, seizures, corneal clouding, vision deficits, cardiomyopathy, and cardiomegaly.  *Age of onset:* 6 months  *Lifespan:* 15 years | Depression.^108^ | Developmental regression, intellectual disability, and exaggerated startle response to loud noises. |
| GM 2 Gangliosidosis (Tay Sachs) | Enzyme: β-hexosaminidase A  Major organs: CNS | Head tremors, cerebellar ataxia, vision deficits, incoordination, and goose stepping gait.^109^  *Age of onset:* 6-9 months  *Lifespan:* 2 years | Motor deficits, slow growth, hypotonia, vision deterioration, muscle spasticity, developmental arrest, and seizures.  *Age of onset*: 3 - 6 months  *Lifespan:* 3 – 5 years | Altered mental status,^109^ dull mentation, and decreased responsiveness to verbal cues.^107^ | Cognitive regression, increased startle response to loud noise, and dementia. |
| GM 2 Gangliosidosis (Sandhoff) | Enzyme(s): β-hexosaminidase A and β-hexosaminidase B or Activator protein  Major organs: CNS | Generalized tremors, cerebellar ataxia, vision deficits, incoordination, stiff gait, and low tail carriage.^110,111^  *Age of onset*: 3 - 9 months  *Lifespan*: 2 years | Motor deficits, slow growth, hypotonia, vision deterioration, muscle spasticity, developmental arrest, seizures, hepatosplenomegaly, heart murmurs, seizures, and dysostosis multiplex.  *Age of onset*: 3 – 6 months  *Lifespan*: 3 – 4 years | Decreased responsiveness to verbal cues, loss of house training, anxiety, decreased appetite, panting, intolerance to loud noise, and trance like behavior.^110,111^ | Decreased responsiveness, cognitive regression, increased startle response to loud noise, and dementia. |
| Neuronal ceroid lipofuscinosis 1 | Enzyme: Palmitoyl protein thioesterase 1  Major organs involved: CNS, heart, endothelial cells, retina | Incoordination, cerebellar ataxia, kyphosis, stiff gait, blindness, and tremors.^112-114^  *Genetic mutation found for*: Miniature Dachshund^113^ and Cane Corso^114^  *Age of onset:* 6-7 months  *Lifespan*: ~14 months | Microcephaly, hypotonia, intellectual and motor disability, ataxia, limited speech, repetitive hand movements, myoclonus, epilepsy, feeding difficulties, and vision loss.  *Age of onset:* 6-24 months  *Lifespan*: 2 to 9 years | Increased anxiety, loss of learned behaviors, decreased interactions with housemate (dogs), dementia, sensitivity to noise, circling, and vocalization.^107,112,113^ | Irritability, developmental regression, and loss of interest in play. |
| Neuronal ceroid lipofuscinosis 2 | Enzyme: pepstatin-sensitive protease, tripeptydylpeptidase  Major organs involved: CNS, retina | Vomiting, diarrhea, weakness, ataxia, and vision deficits^112,115^  *Genetic mutation found for:* Miniature and Standard Dachshund  *Age of onset*: 9 months  *Lifespan*: 12 - 21 months | Epilepsy, ataxia, myoclonus, blindness, impaired walking, speech, and sitting.  *Age of onset*: 2 – 4 years  *Lifespan:* Teenage years | Loss of learned behaviors, hyperactivity, howling, aggression, and circling.^107,115^ | Developmental regression, cognitive decline, and behavior problems. |
| Neuronal ceroid lipofuscinosis 4A | Enzyme: Arylsulfatase G  Major organs involved: CNS, musculoskeletal | Stiff gait, vision deficits, incoordination, heat tremors, ataxia, and general weakness.^116^  *Genetic mutation found for:* American Pit Bull Terrier; American Staffordshire Terrier  *Age of onset*: 3 – 5 years  *Lifespan*: humane euthanasia due to progression within months of clinical signs | Seizures, myoclonic epilepsy, ataxia, incoordination, tremors, and speech difficulty  *Age of onset*: 30 years  *Lifespan:* 45 years | Mental dullness, and dementia.^116^ | Dementia. |
| Neuronal ceroid lipofuscinosis 5 | Enzyme: transmembrane protein  Major organs involved: CNS, heart, endothelial cells, retina | Incoordination, tremors, seizures, vision deficits, weakness, and abnormal gait.^117,118^  *Genetic mutation found for*: Border Collie, Australian Cattle Dog, Golden Retriever  *Age of onset:* 1 year  *Lifespan:* 2 – 3 years | Incoordination, seizures, myoclonic epilepsy, ataxia, blindness  *Age of onset*: 5 years  *Lifespan*: mid-adulthood | Loss of learned behaviors, social anxiety, increased startle response to loud noises, aggression, and agitation.^117,118^ | Developmental regression, and cognitive decline. |
| Neuronal ceroid lipofuscinosis 6 | Enzyme: endoplasmic reticulum transmembrane protein  Major organs involved: CNS, heart, endothelial cells | Progressive incoordination, muscle weakness, abnormal gait, vision loss, difficulty finding food and eating.^119^  *Genetic mutation found for*: Australian Shepherd  *Age of onset*: 1.5 years  *Lifespan*: 2 years | Epilepsy, ataxia, myoclonus, impaired speech, blindness  *Age of onset*: early and late childhood  *Lifespan*: early adulthood | Circling, anxiety, lack of response to owner voices, unwilling to go outside, and increased startle response to sound and touch.^119^ | Developmental regression, and cognitive decline. |
| Neuronal ceroid lipofuscinosis 7 | Enzyme: transmembrane protein  Major organs involved: CNS | Progressive ataxia, vision and olfaction deficits, and seizures.^120^  *Genetic mutation found for*: Chinese Crested Dog; Chihuahua  *Age of onset*: 13 – 21 months  *Lifespan*: 2 years | Epilepsy, blindness, myoclonus, ataxia, and speech difficulties.  *Age of onset:* 2 – 7 years  *Lifespan*: teenage years | Aggression, decreased sense of smell, and less responsive to owners.^120^ | Developmental regression, cognitive decline. |
| Neuronal ceroid lipofuscinosis 8 | Enzyme: transmembrane protein  Major organs involved: CNS | Stiff gait, progressive blindness, incoordination, ataxia, general weakness, epilepsy, and muscle spasms.^121^  *Affected dog breeds*: English Setter, Irish Setter, Gordon Setter, Saluki; Australian Shepherd; Aplenlandische Dachsbracke  *Age of onset:* 14-18 months  *Lifespan*: 2 years | Epilepsy, incoordination, vision and speech loss.  *Age of onset*: 5 – 10 years  *Lifespan*: childhood to adulthood (depending on severity) | Cognitive deficits, and mental dullness.^121^ | Cognitive decline. |
| Neuronal ceroid lipofuscinosis 10 | Enzyme: Cathepsin D  Major organs involved: CNS, liver | Progressive ataxia, hypermetria, incoordination, and wide base stance.^122^  *Affected dog breed*: American Bulldog  *Age of onset*: 1 - 3 years  *Lifespan*: 3.5 – 5.5 years | Muscle rigidity, respiratory failure, seizures, microcephaly, incoordination  *Age of onset*: after birth OR late infancy into adulthood  *Lifespan*: hours to weeks after birth OR shortened depending on occurence | Cognitive decline.^122^ | Cognitive and emotional deficits. |
| Neuronal ceroid lipofuscinosis 12 | Protein: truncated ATP13A2 gene  Major organs involved: CNS | Incoordination, ataxia, vision deficits in dim light, and seizures.^123^  *Affected dog breed*: Tibetan terrier  *Age of onset*: 4 - 6 years  *Lifespan*: 7 - 10 years | Suspected to be a form of Kufor-Rakeb syndrome in humans characterized by parkinsonism and pyramidal dysfunction | Aggression, anxiety, and loss of learned responses.^123^ | Behavior changes, and cognitive decline. |
| Sphingomyelinosis A (Niemann-pick Type A) | Enzyme: sphingomyelinase  Major organs involved: CNS, liver, spleen, bone marrow | Incoordination, exaggerated gait, and hepatosplenomegaly.^124^  *Age of onset*: 2 to 5 months  *Lifespan*: within weeks of clinical signs | Hepatosplenomegaly, ascites, jaundice, feeding problems, constipation, nausea, vomiting, gastrointestinal reflux, loss of reflexes, progressive hypotonia, and recurrent respiratory infections.  *Age of onset*: 9-12 months  *Lifespan*: 3 years | Mental dullness.^124^ | Developmental regression, and irritability. |

*Note: Reprinted [adapted] from Casal, M.L.; Haskins, M.E. List of animal models with mucopolysaccharidosis. In *Mucopolysaccharidosis Update* (Metabolic Diseases – Laboratory and Clinical Research).; Tomatsu, S.; Lavery, C.; Giugliani, R.; Harmatz, P.; Scarpa, M.; Wegrzyn, G.; Orii, T.O.; Eds.; Nova Science Publisher: Hauppauge, NY, USA, 2019; pp. 697-712 [96].

1. NIH National Center for Advancing Translational Sciences. Genetic and rare diseases information center. <https://rarediseases.info.nih.gov/diseases> (accessed on 8/7/19).
2. NIH US National Library of Medicine. Genetics home reference. <https://ghr.nlm.nih.gov/condition/> (accessed on 8/7/19).
3. National MPS Society. MPS Diseases. <http://www.mpssociety.org> (accessed on 8/7/19).
4. Winner, L.K.; Marshall, N.R.; Jolly, R.D.; Trim, P.J.; Duplock, S.K.; Snel, M.F.; Hemsley, K.M. Evaluation of disease lesions in the developing canine mps IIIA brain. *JIMD Rep* 2019, 43, 91-101.
5. Jolly, R.D.; Dittmer, K.E.; Garrick, D.J.; Chernyavtseva, A.; Hemsley, K.M.; King, B.; Fietz, M.; Shackleton, N.M.; Fairley, R.; Wylie, K. 2019. Beta-mannosidosis in german shepherd dogs. *Vet Pathol* 2019, 56, 1-6.
6. Kondagari, G.S.; Ramanathan, P.; Taylor, R. Canine fucosidosis: a neuroprogressive disorder. *Neurodegenerative Dis* 2011, 8, 240-51.
7. Bradbury, A.M.; Bagel, J.H.; Jiang, X.; Swain, G.P.; Prociuk, M.L.; Fitzgerald, C.A.; O’donnell, P.A.; Braund, K.G.; Ory, D.S.; Vite, C.H. Clinical, electrophysiological, and biochemical markers of peripheral and central nervous system disease in canine globoid cell leukodystrophy (krabbe disease). *J Neurosci Res* 2016, 94, 1007-1017.
8. Veterinary Genetic Services. VetGen 2019. <https://www.vetgen.com/canine-NCL.html> (accessed on 8/7/19).
9. Hartley, W.J.; Blakemore, W.F. Neurovisceral glucocerebroside storage (Gaucher’s disease) in a dog. *Vet Pathol* 1973, 10, 191-201.
10. Walvoort, H.C. Glycogen storage disease type II in the Lapland dog. *Vet Q* 1985, 7, 187-90.
11. Animal Labs. Inovagen 2016. [http://www.animalabs.com](http://www.animalabs.com/?s=Spingomylinosis+A+&lang=en) (accessed on 7/22/19).
12. Hasegawa, D.; Yamato, O.; Nakamoto, Y.; Ozawa, T.; Yabuki, A.; Itamoto, K.; Kuwabara, T.; Fujita, M.; Takahashi, K.; Mizoguchi, S.; Orima, H. Serial MRI features of canine GM1 gangliosidosis: a possible imaging biomarker for diagnosis and progression of the disease. *ScientificWorldJournal* 2012: 250197.
13. Sanders, D.N.; Zeng, R.; Wenger, D.A.; Johnson, G.S.; Johnson, G.C.; Decker, J.E.; Katz, M.L.; Platt, S.R.; O’brien, D.P. GM2 gangliosidosis associated with a HEXA missense mutation in Japanese Chin dogs: a potential model for tay sachs disease. *Mol Genet Metab* 2013, 108, 70-75.
14. Kolicheski A, Johnson GS, Villani NA, O’brien, D.P.; Mhlanga-Mutangadura, T.; Wenger, D.A.; Mikoloski, K., Eagleson, J.S.; Taylor, J.F.; Schnabel, R.D.; Katz, M.L. GM2 gangliosidosis in shiba inu dogs with an in-frame deletion in HEXB. *J Vet Intern Med* 2017, 31, 1520-26.
15. Matsuki, N.; Yamamoto, O.; Kusuda, M.; Maede, Y.; Tsujimoto, H.; Ono, K. Magnetic resonance imaging of GM2 gangliosidosis in a golden retriever. *Can Vet J* 2005, 46, 275-278.
16. Katz, M.L.; Rustad, E.; Robinson, G.O.; Whiting, R.E.H.; Student, J.T.; Coates, J.R.; Narfstrom, K. Canine neuronal ceroid lipofuscinoses: promising models for preclinical testing of therapeutic interventions. *Neurobiol Dis* 2017, 108, 277-87.
17. Sanders, D.N.; Farias, F.H.; Johnson, G.S.; Chiang, V.; Cook, J.R.; O’brien, D.P.; Hofmann, S.L.; Lu, J.Y.; Katz, M.L. A mutation in canine ppt1 causes early onset neuronal ceroid lipofuscinosis in a dachshund. *Mol Genet Metab* 2010, 100, 349-56.
18. Kolicheski, A.; Barnes Heller, H.L.; Arnold, S.; Schnabel, R.D.; Taylor, J.F.; Knox, C.A.; Mhlanga-Mutangadura, T.; O’brien, D.P.; Johnson, G.S.; Dreyfus, J.; Katz, M.L. Homozygous PPT1 splice donor mutation in a cane corso dog with neuronal ceroid lipofuscinosis. *J Vet Intern Med* 2017, 31, 149-157.
19. Awano, T.; Katz, M.L.; O’Brien, D.P.; Sohar, I.; Lobel, P.; Coates, J.R.; Khan, S.; Johnson, G.C.; Giger, U.; Johnson, G.S. A frame shift mutation in canine TPP1 (the ortholog of human CLN2) in a juvenile dachshund with neuronal ceroid lipofuscinosis. *Mol Genet Metab* 2006, 89, 254-60.
20. Abitbol, M.; Thibaud, J.L.; Olby, N.J.; Hitte, C.; Puech, J.P.; Maurer, M.; Pilot-Storck, F.; Hedan, B.; Dreano, S.; Brahimi, S.; Delattre, D.; Andre, C.; Gray, F.; Delisle, F.; Caillaud, C.; Bernex, F.; Panthier, J.J.; Aubin-Houzelstein, G.; Blot, S.; Tiret, L. A canine arylsulfatase G (ARSG) mutation leading to a sulfatase deficiency is associated with neuronal ceroid lipofuscinosis. *Proc Natl Acad Sci U S A* 2010, 107, 14775-14780.
21. Gilliam, D.; Kolicheski, A.; Johnson, G.S.; Mhlanga-Mutangadura, T.; Schnabel, R.D.; Katz, M.L. Golden retriever dogs with neuronal ceroid lipofuscinosis have a two base pair deletion and frameshift in CLN5. *Mol Genet Metab* 2015, 115, 101-109.
22. Kolicheski, A.; Johnson, G.S.; O’Brien, D.P.; Mhlanga-Mutangadura, T; Gilliam, D.; Guo, J.; Anderson-Sieg, T.D.; Schnabel, R.D.; Taylor, J.F.; Lebowitz, A.; Swanson, B.; Hicks, D.; Niman, Z.E.; Wininger, F.A.; Carpentier, M.R.; Katz, M.L. Australian cattle dogs with neuronal ceroid lipofuscinosis are homozygous for a CLN5 nonsense mutation previously identified in border collies*. J Vet Intern Med* 2016, 30, 1149-1158.
23. Katz, M.L.; Farias, F.H.; Sanders, D.N., Zeng, R.; Khan, S.; Johnson, G.S.; O’brien, D.P. A missense mutation in canine CLN6 in an Australian shepherd with neuronal ceroid lipofuscinosis. *J Biomed Biotechnol* 2011, 2011: 198042.
24. Ashwini, A.; D’Angelo, A; Yamato, O.; Giordano, C.; Cagnotti, G.; Harcourt-Brown, T.; Mhlanga-Mutangadura, T.; Guo, J.; Johnson, G.S.; Katz, M.L. Neuronal ceroid lipofuscinosis associated with MFSD8 mutation in chihuahuas. *Mol Genet Metab* 2016, 118, 326-332.
25. Katz, M.L.; Khan, S.; Awano, T.; Shahid, S.A.; Siakotos, A.N.; Johnson, G.S. A mutation in the CLN8 gene in English setter dogs with neuronal ceroid lipofuscinosis. *Biochem Biophys Res Commun* 2005, 327, 541-547.
26. Awano, T.; Katz, M.L.; O’Brien, D.P.; Taylor, J.F.; Evans, J.; Khan, S.; Sohar, I.; Lobel, P.; Johnson, G.S. A mutation in the cathepsin D gene (CTSD) in American bulldogs with neuronal ceroid lipofuscinosis. *Mol Genet Metab* 2006, 87, 341-348.
27. Farias, F.H.; Zeng, R.; Johnson, G.S.; Wininger, F.A.; Taylor, J.F.; Schnabel, R.D.; McKay, S.D.; Sanders, D.N.; Lohi, H.; Seppala, E.H.; Wade, C.M.; Lindblad-Toh, K.; O’brien, D.P.; Katz, M.L. A truncating mutation in ATP13A2 is responsible for adult onset neuronal ceroid lipofuscinosis in Tibetan terriers. *Neurobiol Dis* 2011, 42, 468-474.
28. Bundza, A.; Lowden, J.A.; Charlton, K.M. Niemann pick disease in a poodle dog. *Vet Pathol* 1979, 16, 530-538.

Databases searched: Online Mendelian Inheritance in Animals (OMIA); Google Scholar

Terms used in search functions: Lysosomal storage disease, MPS, mannosidosis, fucosidosis, globoid cell dystrophy, glucocerebrosidosis, glycogen storage disease, gangliosidosis, neuronal ceroid lipofuscinosis, sphingomyelinosis

Table S2: Causes for euthanasia of dogs during the study.

| **ID #** | **Sex** | **Group** | **Age of Euthanasia (weeks)** | Reason for euthanasia |
| --- | --- | --- | --- | --- |
| 672 | Male | MPS I untreated | 52 | Umbilical hernia strangulating intestine |
| 690 | Male | MPS I treated | 68 | Testicular torsion |
| 725 | Male | MPS I untreated | 57 | Cervical cord compression with progressive hind limb weakness and ataxia |
| 726 | Male | MPS I untreated | 60 | Cervical cord compression with progressive hind limb weakness and ataxia |
